# Associations between Influenza Vaccination and Health Care Access among Adults in the United States

**DOI:** 10.3390/vaccines11020416

**Published:** 2023-02-11

**Authors:** Morgan Gurel-Headley, Mariam Mamisashvili, Sheena CarlLee, Sharon Reece, Christina Chapman, Shashank Kraleti, Jennifer A. Andersen, James P. Selig, Don E. Willis, Ji Li, Pearl A. McElfish

**Affiliations:** 1 College of Medicine, University of Arkansas for Medical Sciences, 4301 W. Markham St., Little Rock, AR 72205, USA; mpgurel@uams.edu (M.G.-H.); skraleti@uams.edu (S.K.); 2 Fay W. Boozman College of Public Health, University of Arkansas for Medical Sciences, 4301 W. Markham St., Little Rock, AR 72205, USA; 3 American MD Program, Faculty of Medicine, Tbilisi State Medical University, 33 Vazha Pshavela Ave., 0162 Tbilisi, Georgia; m.mamisashvili@tsmu.edu.ge; 4 College of Medicine, University of Arkansas for Medical Sciences Northwest, 1125 N. College Ave., Fayetteville, AR 72703, USA; swcarllee@uams.edu (S.C.); screece@uams.edu (S.R.); cechapman@uams.edu (C.C.); 5 College of Medicine, University of Arkansas for Medical Sciences Northwest, 2708 S. 48th St., Springdale, AR 72762, USA; jaandersen@uams.edu (J.A.A.); dewillis@uams.edu (D.E.W.); 6 Fay W. Boozman College of Public Health, University of Arkansas for Medical Sciences Northwest, 2708 S. 48th St., Springdale, AR 72762, USA; jpselig@uams.edu (J.P.S.); jli2@uams.edu (J.L.)

**Keywords:** influenza, influenza vaccination, health care access, racial discrimination

## Abstract

Few studies have investigated the relationship between influenza vaccination and health care access. Furthermore, despite the well-documented disparities in vaccine coverage for communities of color, few studies have examined how experiences of discrimination may influence vaccine uptake. To fill this gap in the literature, this study examined associations between 5-year influenza vaccination rates and sociodemographic characteristics, health care access, and racial discrimination. Age, race/ethnicity, education, health care coverage, primary care provider, no medical care due to cost, and routine doctor checkups were significant correlates of 5-year influenza vaccination. In contrast to previous studies, discrimination scores were not a significant correlate of regular influenza vaccination. Respondents who reported forgoing care due to cost were less likely to report vaccination every year out of the last 5 years compared to all of the less frequent categories combined, demonstrating a more complex association between sometimes not being able to afford medical care and influenza vaccination. Future research should examine the relationship between influenza vaccination uptake, racial discrimination, and forgone care due to cost to enhance resources and messaging for influenza vaccination uptake.

## 1. Introduction

The Centers for Disease Control and Prevention (CDC) estimates that the influenza virus infected nearly 40 million people in the United States (US) during the 2019–2020 season, causing 400,000 hospitalizations and over 20,000 deaths [1]. The estimated economic burden of influenza to the US health care system is USD 11.2 billion annually [2]. The influenza vaccine is the most effective form of protection against the virus, its complications, and associated costs [3]. The COVID-19 pandemic has only heightened the importance of influenza vaccination, as health care professionals have expressed concerns about possibilities of a “twindemic” or “tridemic” of respiratory viruses [4].

Although uptake of the vaccine has increased for adults over the past three influenza seasons, only 50.2% of the US adult population was vaccinated during the 2020–2021 season [3]. The existing literature shows that rates of influenza vaccination vary by several sociodemographic factors, including age, gender, race/ethnicity, education, and income. Individuals who are older, are women [5,6,7,8], have a higher level of education [6,7], and have higher income [7] are more likely to get the influenza vaccine [9]. Racial and ethnic disparities in vaccine coverage persist, with communities of color being less likely to receive the influenza vaccine [5,7,9,10,11,12,13,14,15].

Though associations between sociodemographic factors and influenza vaccination uptake have been examined, few studies have investigated the relationship between influenza vaccination and health care access [6,16]. Furthermore, despite the well-documented disparities in vaccine coverage for communities of color, few studies have examined how experiences of discrimination may influence vaccine uptake [17,18,19]. Race is “a system of structuring opportunity and assigning value based on the social interpretation of how one looks” [20,21]. However, medical researchers rarely recognize race as a social, rather than biological, construct [22,23]. In doing so, key mechanisms generating racial health disparities—namely, forms of racism—are overlooked [24]. Racial discrimination is unfair treatment based on one’s perceived race, ethnicity, or skin color and is a form of personally mediated racism [25]. The few studies that have investigated relationships between experiences of racial discrimination and influenza vaccination have been limited by their use of single-item measures focused primarily on medical settings, rather than considering the influence of discrimination across multiple settings [17,18,19]. Despite the limitations of the measures used, these studies have documented associations between influenza vaccination and experiences of racial discrimination [17,18,19]. The first known study to document the association found respondents who experienced discrimination had half the uptake as those with no experiences of discrimination [19]. Subsequent studies supported this finding and found evidence that it may be mediated by higher perceived vaccine risk [17,18].

To fill this gap in the literature, this study undertook the following aims. First, the study examined 5-year influenza vaccination rates among US adults by sociodemographic characteristics (age, gender, education, marital status, and race/ethnicity). Based on prior literature, it was hypothesized that being older, identifying as a woman, being married/cohabiting, and having a higher education would be positively associated with influenza vaccination; minority race/ethnicity was hypothesized to be negatively associated with influenza vaccination. Second, the study examined associations between health care access and influenza vaccination. This study used multiple components of health care access to examine this relationship. It was hypothesized that having health care insurance coverage, having a primary care provider, and having seen a provider for a routine checkup within the past 2 years would be positively associated with influenza vaccination; it was hypothesized that forgone care due to costs would be negatively associated with influenza vaccination. Finally, this study investigated associations between influenza vaccination and respondent-reported experiences of racial discrimination in a range of settings. It was hypothesized that a higher racial discrimination score would be negatively associated with influenza vaccination.

## 2. Methods

### 2.1. Procedures

Between 7 September 2021 and 3 October 2021, potential respondents were recruited via email using an online registry of research volunteers across the US. Data were obtained through an online survey available in both English and Spanish. To meet inclusion criteria, respondents had to be aged 18 years or older and currently residing in the US. The following study information was provided to each potential respondent in the recruitment emails: (1) estimated study duration; (2) potential risks and benefits of participation; (3) voluntary nature of participation; and (4) confidential nature of participation and data. Respondents provided consent by agreeing to participate in the survey. Data were obtained from a total of 2022 participants who completed the survey. Study procedures were reviewed and approved by the University of Arkansas for Medical Sciences Institutional Review Board (IRB #263020).

To ensure broad representation, racial and ethnic minority populations, including Asian, Black, Hispanic/Latinx, American Indian or Alaska Native (AIAN), and Native Hawaiian or Pacific Islander (NHPI), were intentionally oversampled. Oversampling allows for disaggregation of data across racial and ethnic groups, which in turn aids in revealing diverse attitudes and lived experiences [26,27]. The data were weighted to represent the US population using the random iterative method [28]; this was done across demographic variables including gender (man, woman, non-binary), race/ethnicity (Asian, Black, Hispanic/Latinx, AIAN, NHPI, and White), and age (18–24, 25–34, 35–44, 45–54, 55–64, 65+).

### 2.2. Measures

#### 2.2.1. Influenza Vaccine Uptake

The dependent variable was an ordinal measure of 5-year influenza vaccine uptake. Participants responded to the question, “How many years in the past 5 years have you gotten a seasonal flu vaccine?” Response options included “never,” “1–2 years,” “3–4 years,” and “every year."

#### 2.2.2. Sociodemographic Characteristics and Experiences with Discrimination

Age, gender, race/ethnicity, education, marital or cohabitating status, and experiences of discrimination were measured as possible correlates of vaccine uptake. Participants’ reported year of birth was used to calculate age. Gender was reported as either man or woman. Options of non-binary and self-identification were available, but too few participants selected these options (N = 8; 0.4%) to be included in the analysis. As a result, these responses were coded as missing. Participants reported their race/ethnicity, and responses were grouped into: Asian, Black, Hispanic/Latinx, AIAN, NHPI, or White. Participants reported their highest level of school completed, and responses were grouped into: high school degree/graduate equivalency degree (GED) or lower, some college/associate degree, and bachelor’s degree or higher. Participants reported their marital or cohabitating status with responses grouped into two categories: married or cohabitating and unmarried or single. Participants were asked if they had experienced discrimination because of their race, ethnicity, or color in any of the following situations: (1) at school; (2) when being hired for a job; (3) at work; (4) in securing housing; (5) in receiving medical care; (6) at a store or restaurant; (7) when securing credit or applying for a mortgage; (8) on the street/in public; and (9) from police or by the courts. Response options for each situation included (1) never; (2) once; (3) two or three times; and (4) four or more times. Item responses were recoded into values of 0, 1, 2.5, and 5 [29]. Discrimination scores were calculated as the sum of the item scores for each of the nine discrimination situations. This sum score could range from a minimum of 0 to a maximum of 45.

#### 2.2.3. Health Care Access

Health care access was measured using four survey items: (1) “Do you have any kind of health care coverage, including health insurance, prepaid plans, such as HMOs, government plans, such as Medicare, or Indian Health Service?” (Yes/No); (2) “Do you have one person you think of as your personal doctor or health care provider?” (Yes/No); (3) “About how long has it been since you last visited a doctor for a routine checkup?” Response options included: in the past year, in the past 2 years, in the past 5 years, 5 or more years ago, and never; and (4) “Was there a time in the past 12 months when you needed a doctor but could not see one because of the cost?” (Yes/No). Due to potential changes in health care-seeking behaviors due to the COVID-19 pandemic, responses to the doctor visit/routine checkup item were collapsed into two categories: within the past 2 years and more than 2 years ago.

### 2.3. Statistical Analyses

Data were cleaned and analyzed using SAS 9.4 and RStudio [30]. No duplicate records were detected. Participants with incomplete responses (N = 75; 3.7%) were omitted from the analyses. Health care coverage was the variable most often missing (N = 69). Weights, based on population totals, were used to make the results better represent population results. Weights were computed using the random iterative method across the demographic variables of age (18–24, 25–34, 35–44, 45–54, 55–64, 65+), gender (man, woman, non-binary, and self-described), and race/ethnicity (Asian, Black, Hispanic/Latinx, AIAN, NHPI, and White). A cumulative logit model [31] was used because of the ordinal nature of the 5-year influenza vaccine uptake variable. This model consists of three cumulative logits that map onto three cumulative probabilities for past 5-year vaccination: (1) the probability of vaccination every year, 3–4 years, or 1–2 years vs. never; (2) the probability of vaccination every year or 3–4 years vs. 1–2 years or never; and (3) the probability of vaccination every year vs. 3–4 years, 1–2 years, or never. For correlates meeting the proportional odds assumption, a single coefficient was used for each of the cumulative logits. When the assumption was not met, separate coefficients were estimated for each of the cumulative logits. The model was structured to estimate the probability of more regular influenza vaccination. Using the R package brant [32], a Brant test was conducted to assess the assumption of proportional odds. The assumption of proportional odds was not met for at least one of the independent variables (χ^2^ = 134.03 with df = 34; *p* <.0001). The results showed both age (*p* < 0.0001) and forgone care due to cost (*p* < 0.0001) violated the proportional odds assumption. Therefore, a partial proportional odds (PPO) regression was used, with separate slopes for each cumulative logit for age and no care due to cost but single slopes for all other correlates.

## 3. Results

### 3.1. Descriptives

A total of 2022 individuals completed at least part of the survey. Seventy-five (3.7%) individuals were excluded from the analyses because of one or more missing responses. Table 1 provides weighted estimates of descriptive statistics and unweighted sample sizes for all variables included in the regression model for this study.

### 3.2. Partial Proportional Odds Cumulative Logistic Regression

Table 2 provides the results of the partial proportional odds regression analysis. Age, race/ethnicity, education, health care coverage, primary care provider, no medical care due to cost, and routine doctor checkups were statistically significant correlates of 5-year influenza vaccination. No statistically significant associations were found between 5-year influenza vaccination and gender, marital status, or experiences of racial discrimination.

### 3.3. Sociodemographic Correlates

With only one exception, younger respondents (18–29, 30–44, and 45–59) had lower odds of regular influenza vaccination than those aged 60+. The one exception was for the model comparing never vaccinated with 1–2 years or more frequent vaccination, where those aged 18–29 were not significantly different from those aged 60+. Asian participants had 69% higher odds of regular influenza vaccination than White participants (OR = 1.69; CI = 1.24, 2.31). Black participants had 37% lower odds of regular vaccination than White participants (OR = 0.63; CI = 0.49, 0.81), and AIAN participants had 38% lower odds than White participants (OR = 0.62; CI = 0.40, 0.96). Those with some college or an associate degree had 23% lower odds of regular influenza vaccination than participants with a high school diploma, GED, or less (OR = 0.77; CI = 0.62, 0.96).

### 3.4. Health Care Access Correlates

Participants with health care coverage had 43% higher odds of regular influenza vaccination than those without health care coverage (OR = 1.43; CI = 1.08, 1.89). Those who reported having a primary care provider had 64% higher odds of regular influenza vaccination than those without a primary care provider (OR = 1.64; CI = 1.26, 2.14). Participants who reported having visited a doctor in the last 2 years had over twice the odds of regular influenza vaccination compared to those who had not seen a doctor in the past 2 years (OR = 2.93; CI = 2.26, 3.78). The association between having forgone medical care due to cost and regular influenza vaccination was complex. Participants reporting not seeing a doctor due to cost had higher odds of *any* influenza vaccination (every year, 3–4 years, or 1–2 years) relative to never receiving an influenza vaccination (OR = 1.58; 1.17, 2.13), but had lower odds of influenza vaccinations every year relative to less regular vaccination (3–4 years, 1–2 years, or never) (OR = 0.65; 0.48, 0.88).

## 4. Discussion

This study examined the association between sociodemographic factors, health care access, and experiences of discrimination on influenza vaccine uptake. Regarding sociodemographic factors, we hypothesized that those who identify as women, are younger, and have lower educational attainment would be less likely to receive the influenza vaccine. Gender was not a significant factor for receiving the influenza vaccine. Respondents aged 18–59 had lower odds of regular influenza vaccination than those aged 60 and older, with one exception: when comparing never vaccinated vs. all other categories, those aged 18–29 were not significantly different from those aged 60+. This finding is mostly consistent with prior literature that has found older individuals to be more likely to receive the influenza vaccine [3,6,9]. Results regarding education were mixed and somewhat unexpected [6,9]. Those with some college/an associate degree were less likely than those with a high school diploma/GED to receive the influenza vaccine. However, the result for those with a bachelor’s degree or higher was not significant.

Regarding race and ethnicity, Asian respondents were approximately two-thirds more likely to receive the influenza vaccine than White respondents. This is consistent with prior studies demonstrating Asian Americans are more likely to be vaccinated than members of other racial and ethnic groups [33]. Black and AIAN respondents were less likely than White respondents to receive the influenza vaccine. These findings are also congruent with the established literature [34,35,36,37,38]. Although NHPI respondents reported slightly lower vaccination uptake than White respondents, the difference was not statistically significant. A CDC report [39] on health care access and utilization among NHPI found that they were more likely to have received an influenza vaccination in the past year compared to the general US population and that their vaccination coverage was similar to that of Asian Americans.

This study makes a necessary contribution to the literature, as it is one of the few national studies with a large sample of AIAN respondents. Furthermore, it is one of the first studies to have a large number of disaggregated Asian and NHPI respondents. Often, these groups are combined in analyses, which impedes the potential to recognize unique experiences and outcomes. These results further demonstrate the need to disaggregate data on Asian and NHPI individuals [27], as differences were found between Asian and White respondents but not between NHPI and White respondents.

We hypothesized that participants with a primary care provider, a checkup with their doctor within the last 2 years, and health insurance coverage would be more likely to receive the influenza vaccine. We also hypothesized that those who had forgone medical care due to cost would be less likely to receive the influenza vaccine. The results supported both these hypotheses. Respondents with a primary care provider were more likely to receive the influenza vaccine than those without, and those who had health insurance coverage were more likely to receive the influenza vaccine than those who did not. Individuals who had seen a doctor for a checkup within the last 2 years were nearly three times more likely to receive the influenza vaccine than those who had not. These findings align with developing literature demonstrating the value of health care providers in vaccine uptake [6,26,40,41,42].

Results of the relationship with forgone care due to cost were mixed. Individuals who had forgone care due to cost were more likely than those who had not forgone care to receive the influenza vaccine at least once in the last 5 years (every year, 1–2 years, 3–4 years) than not at all. However, respondents who had forgone care were less likely to report vaccination every year out of the last 5 years compared to all of the less frequent categories combined (never, 1–2 years, 3–4 years) [9]. Those who face cost barriers may forgo an influenza vaccination even if the vaccines themselves are free of charge. For example, if an individual has vaccine-related questions or concerns but is unable to afford a visit with their provider, they may be discouraged from receiving the influenza vaccine. These results suggest a more complex association between sometimes not being able to afford medical care and influenza vaccination, such that those forgoing care were less likely to be vaccinated every year but were more likely to be vaccinated at least once in the past 5 years.

We hypothesized that respondents who reported more experiences of racial discrimination would be less likely to receive the influenza vaccine; however, racial discrimination was not a significant correlate of regular influenza vaccination. These findings contrast with some reports in the literature that found negative associations between experiences of discrimination and influenza vaccination [18,19]. Discrepancies between our findings and past research may be due to differences in measurement or other analytical decisions. For example, both prior studies used measures of discrimination specific to medical/health care settings [18,19], while we employed a standard multi-item measure of discrimination across many settings [29]. Given that prior studies that found a relationship utilized measures of discrimination specific to medical and health care settings, it may be that our broader measure of racial discrimination is less salient for influenza vaccination than those that are health care-specific. Further, Quinn and colleagues [18] limited their analysis to White and African American respondents in the US, while we examined the association across many racial and ethnic groups. The measure we utilized may not capture experiences of discrimination that are more common among this more diverse sample. For example, it does not include discrimination based on language or accent [43,44].

Although we did not find a significant association between influenza vaccination and this particular form of personally mediated racism, internalized or structural racism may be more salient for vaccine uptake, as some research has demonstrated for COVID-19 vaccination [45]. Importantly, failure to meet the threshold for statistical significance should not be interpreted to mean no relationship exists—we simply did not find evidence for a relationship in these data. To improve health disparities related to vaccination, it is critical to study the impact of all forms of racism on vaccination rates. Our study is one step in this necessary investigation. Future research should continue to examine the role that racism at varying levels may play in shaping influenza vaccination.

### Limitations

The results reported should be considered with some limitations in mind. The data are cross-sectional, and our analyses, in turn, could not establish temporal order or causal relationships. Although we drew on a large national sample and utilized weights, our sampling methods were not random. The non-probability sample further limits the generalizability of this study. Online surveys provide many advantages; however, they often suffer from low participation among minoritized racial and ethnic groups and those with lower educational attainment. In anticipation of this, we did correct by oversampling minoritized racial and ethnic groups to ensure representation and allow for disaggregation. Further, we relied on self-reported measures of items, and our outcome measure relied on the recall of respondents to remember past vaccination behaviors over the past 5 years. The time frame in which the study was conducted may have also influenced the results. During the fall of 2021, there were concurrent influenza and COVID-19 vaccination efforts that may have influenced influenza vaccine uptake [46]. Finally, although self-reported experiences of discrimination have been found to be associated with a wide range of health outcomes and behaviors, those experiences may be less salient for vaccination uptake than structural racism, which we did not account for in this analysis.

## Figures and Tables

**Table 1:** Weighted and Unweighted Descriptive Statistics for All Study Variables (N = 1947).

Measures	*Weighted % or Mean (SD)*	*Unweighted N*
**Age (N = 2022)**		
18–29	18.9	360
30–44	27.0	716
45–59	23.7	495
60+	30.3	451
**Gender (N = 2014)**		
Man	50.0	952
Woman	50.0	1062
**Race/Ethnicity (N = 2022)**		
Asian	10.0	304
Black	20.0	404
Hispanic/Latinx	20.0	404
AIAN	5.0	254
NHPI	5.0	252
White	40.0	404
**Education (N = 2022)**		
HS degree/GED or lower	28.2	581
Some college/associate degree	34.0	692
Bachelor’s degree or higher	37.8	749
**Marital Status (N = 2022)**		
Married/cohabitating	47.6	976
Unmarried/single	52.4	1046
**Health Care Coverage (N = 1953)**		
Yes	86.9	1675
No	13.1	278
**Primary Care Provider (N = 2022)**		
Yes	81.3	1594
No	18.7	428
**No Medical Care Due to Cost (N = 2022)**		
No	83.4	1634
Yes	16.6	388
**Routine Doctor Checkup (N = 2022)**		
Within the past 2 years	81.3	1624
More than 2 years ago	18.8	398
**5-Year Influenza Vaccination (N = 2022)**		
Never	29.5	619
1–2 years	22.3	489
3–4 years	10.3	209
Every year	37.9	705
**Discrimination Score (N = 2022**)	6.4 (9.8)	

Note: Percentages may not total 100 due to rounding. AIAN = American Indian or Alaska Native; GED = graduate equivalency degree; HS = high school; NHPI = Native Hawaiian or Pacific Islander; SD = standard deviation.

**Table 2:** Weighted Proportional Ordinal Logistic Regression—5-Year Influenza Vaccination (N = 1947).

	*B*	*SE*	*p*	*OR (95% CI)*
**Age**	*Every Year* vs. *All Others*			
18–29	−0.882	0.169	**<0.001**	0.414 (0.298, 0.576)
30–44	−1.143	0.141	**<0.001**	0.319 (0.242, 0.420)
45–59	−0.673	0.132	**<0.001**	0.510 (0.394, 0.661)
60+	-	-	-	-
	*Every Year and 3–4 Years* vs. *All Others*			
18–29	−0.906	0.163	**<0.001**	0.404 (0.293, 0.557)
30–44	−1.109	0.137	**<0.001**	0.330 (0.252, 0.432)
45–59	−0.808	0.134	**<0.001**	0.446 (0.343, 0.579)
60+	-	-	-	-
	*Every Year, 3–4 Years, and 1–2 Years* vs. *Never*			
18–29	0.045	0.178	0.803	1.045 (0.738, 1.482)
30–44	−0.579	0.147	**<0.001**	0.560 (0.420, 0.747)
45–59	−0.521	0.147	**<0.001**	0.594 (0.445, 0.792)
60+	-	-	-	-
**Gender**				
Woman	−0.160	0.088	0.069	0.852 (0.716, 1.013)
Man	-	-	-	-
**Race/Ethnicity**				
Asian	0.525	0.159	**<0.001**	1.690 (1.238, 2.306)
Black	−0.469	0.129	**<0.001**	0.626 (0.486, 0.806)
Hispanic/Latinx	−0.121	0.123	0.326	0.886 (0.697, 1.127)
AIAN	−0.479	0.221	**0.030**	0.619 (0.401, 0.955)
NHPI	−0.038	0.214	0.858	0.962 (0.633, 1.464)
White	-	-	-	-
**Education**				
Bachelor’s degree or higher	−0.100	0.114	0.382	0.905 (0.723, 1.132)
Some college/associate degree	−0.262	0.112	**0.020**	0.769 (0.617, 0.959)
HS diploma/GED or lower	-	-	-	-
**Marital Status**				
Married/cohabitating	0.039	0.091	0.671	1.039 (0.870, 1.241)
Unmarried/single	-	-	-	-
**Health Care Coverage**				
Yes	0.357	0.141	**0.011**	1.429 (1.084, 1.885)
No	-	-	-	-
**Primary Care Provider**				
Yes	0.493	0.136	**<0.001**	1.637 (1.255, 2.135)
No	-	-	-	-
**No Medical Care Due to Cost**	*Every Year* vs. *All Others*			
Yes	−0.427	0.153	**0.005**	0.653 (0.484, 0.880)
No	-	-	-	-
	*Every Year and 3–4 Years* vs. *All Others*			
Yes	−0.194	0.139	0.163	0.823 (0.627, 1.082)
No	-	-	-	-
	*Every Year, 3–4 Years, and 1–2 Years* vs. *Never*			
Yes	0.455	0.153	**0.003**	1.576 (1.168, 2.125)
No	-	-	-	-
**Routine Doctor Checkup**				
Within the past 2 years	1.074	0.131	**<0.001**	2.926 (2.263, 3.783)
More than 2 years ago	-	-	-	-
**Discrimination Score**	0.006	0.005	0.190	1.006 (0.997, 1.016)

Note: Significant *p*-values are in bold. AIAN = American Indian or Alaska Native; Β = beta; CI = confidence interval; GED = graduate equivalency degree; HS = high school; NHPI = Native Hawaiian or Pacific Islander; OR = odds ratio; SE = standard error.

## Data Availability

The deidentified data underlying the results presented in this study may be made available upon request from the corresponding author, Dr. Pearl A. McElfish, at pamcelfish@uams.edu. The data are not publicly available in accordance with funding requirements and participant privacy.
